# Selection of Diagnostically Significant Regions of the *SLC26A4* Gene Involved in Hearing Loss

**DOI:** 10.3390/ijms232113453

**Published:** 2022-11-03

**Authors:** Valeriia Yu. Danilchenko, Marina V. Zytsar, Ekaterina A. Maslova, Olga L. Posukh

**Affiliations:** 1 Federal Research Center Institute of Cytology and Genetics, Siberian Branch of the Russian Academy of Sciences, 630090 Novosibirsk, Russia; 2 Novosibirsk State University, 630090 Novosibirsk, Russia

**Keywords:** hearing loss, *SLC26A4*, pendrin, pathogenic variants, bioinformatic analysis, molecular testing

## Abstract

Screening pathogenic variants in the *SLC26A4* gene is an important part of molecular genetic testing for hearing loss (HL) since they are one of the common causes of hereditary HL in many populations. However, a large size of the *SLC26A4* gene (20 coding exons) predetermines the difficulties of its complete mutational analysis, especially in large samples of patients. In addition, the regional or ethno-specific prevalence of *SLC26A4* pathogenic variants has not yet been fully elucidated, except variants c.919-2A>G and c.2168A>G (p.His723Arg), which have been proven to be most common in Asian populations. We explored the distribution of currently known pathogenic and likely pathogenic (PLP) variants across the *SLC26A4* gene sequence presented in the Deafness Variation Database for the selection of potential diagnostically important parts of this gene. As a result of this bioinformatic analysis, we found that molecular testing ten *SLC26A4* exons (4, 6, 10, 11, 13–17 and 19) with flanking intronic regions can provide a diagnostic rate of 61.9% for all PLP variants in the *SLC26A4* gene. The primary sequencing of these *SLC26A4* regions may be applied as an initial effective diagnostic testing in samples of patients of unknown ethnicity or as a subsequent step after the targeted testing of already-known ethno- or region-specific pathogenic *SLC26A4* variants.

## 1. Introduction

Over 5% of the world’s human population is affected by hearing loss (HL) [1], and more than a half of all HL cases are a result of genetic causes [2]. Hereditary HL represents both nonsyndromic (isolated) forms and several hundred different syndromes, including HL as one of the clinical symptoms. No less than 120 genes are currently known to be implicated in nonsyndromic HL. The loci responsible for different nonsyndromic HL forms and corresponding HL phenotypes are abbreviated DFN (stands for DeaFNess). According to the mode of inheritance, nonsyndromic HL can be classified as autosomal dominant (DFNA), autosomal recessive (DFNB), or X-linked (DFNX) [3]. Autosomal recessive nonsyndromic hearing loss 1A (DFNB1A, OMIM 220290), caused by pathogenic variants in the *GJB2* gene (13q12.11, OMIM 121011), is considered to be the most common form of HL in many global populations [4].

The second common contributor to the etiology of hereditary HL in many global regions, at least in Asian countries, is the *SLC26A4* (Solute carrier family 26, member 4/pendrin, 7q22.3, OMIM 605646) gene [5,6,7,8,9,10]. *SLC26A4* encodes an anion transporter protein known as pendrin (780 amino acids), which belongs to the solute carrier family 26A. The high expression of *SLC26A4* is observed in tissues of the inner ear, thyroid and kidneys. Mutations in the *SLC26A4* gene cause nonsyndromic recessive deafness (DFNB4, OMIM 600791) and Pendred syndrome (PDS, OMIM 274600), which combines sensorineural HL and the enlargement of the thyroid glands (goiter). In the inner ear, pendrin has been shown to be responsible for maintaining the endocochlear potential and fluid homeostasis, whereas in the thyroid, pendrin mediates iodide transport [11,12,13,14,15,16]. The *SLC26A4*-related HL is accompanied by the enlarged vestibular aqueduct (EVA) and other malformations of the inner ear structures. These malformations appear to be due to endolymphatic hydrops as a likely consequence of defects in anion and fluid transport because of the deficiency or dysfunction of pendrin [14,15,16].

The *SLC26A4* gene is located in chromosome 7 (7:107,660,828-107,717,809; GRCh38 assembly), covers about 57 kb of genomic DNA and comprises 21 exons (including the first non-coding exon). To date, the total number of variations identified in the *SLC26A4* gene sequence exceeds 22500 (dbSNP: https://www.ncbi.nlm.nih.gov/snp/?term=SLC26A4, accessed on 1 August 2022). Among them, several hundred variants (including missense, frameshift, splicing site, nonsense and small deletion mutations) are shown to be associated with a wide range of HL phenotypes (the Deafness Variation Database: https://deafnessvariationdatabase.org/gene/SLC26A4, accessed on 6 June 2022; Clin-Var: https://www.ncbi.nlm.nih.gov/clinvar/?term=SLC26A4, accessed on 6 June 2022; the Human Gene Mutation Database: http://www.hgmd.cf.ac.uk/ac/, accessed on 1 February 2022).

Many worldwide studies aimed at assessing the contribution of *SLC26A4* to the HL etiology and studying the genotype–phenotype correlations mediated by the presence of different *SLC26A4* pathogenic variants in patients. Thereby, their screening has become an important part of molecular genetic testing for HL, especially in patients with detected EVA. Numerous studies revealed a diverse spectrum of *SLC26A4* pathogenic variants and their varying prevalence in patients from different regions of the world [5,6,7,9,17,18,19,20]. These facts could be due to the region-specific landscape of the *SLC26A4* gene’s allelic diversity, the heterogeneity in size and phenotypic characteristics of the examined cohorts of patients in different studies, and the variable sensitivity of methods used for the *SLC26A4* analysis. The region- or ethno-specific prevalence of the *SLC26A4* pathogenic variants has not yet been fully elucidated. However, the spectrum of *SLC26A4* pathogenic variants found in Asian populations appears to differ from that in populations of Caucasian origins [9,19]. For example, pathogenic variants c.919-2A>G (previously named IVS7-2A>G) and c.2168A>G (p.His723Arg) are the most common in East Asian populations, whereas they are very rare in Europe [9,19].

The sequential analysis of the *SLC26A4* gene in a particular patient continues until two recessive pathogenic *SLC26A4* variants are detected; therefore, a diagnosis is made. The mutational analysis of the *SLC26A4* gene nucleotide sequence during routine diagnostics is difficult due to the large size of the gene. Various strategies are used to diagnose the *SLC26A4*-associated HL. There is a targeted search (from detection of a single mutation to simultaneous detection of several dozen mutations) of *SLC26A4* variants that are already known as pathogenic (including traditional allele specific oligonucleotide analysis, PCR-RFLP-analysis (Polymerase Chain Reaction-Restriction Fragment Length Polymorphism), different SNP (Single Nucleotide Polymorphism) scan assays and microarray-based technology) or preliminary screenings by SSCP (Single Strand Conformation Polymorphism) or DHPLC (Denaturing High Performance Liquid Chromatography) analysis of such variants followed by confirmations by using direct sequencing [6,21,22,23,24,25,26,27,28,29]. In addition, next-generation sequencing (NGS) techniques, which are capable of detecting both known and novel causative variants in many genes implicated in HL, including *SLC26A4*, are increasingly used for routine diagnostics [30,31]. All these approaches are also widely used in different multi-step hierarchical screenings specifically designed for a particular population or region. Each of these diagnostic methods has its own advantages and limitations, but in any case, direct sequencing is considered necessary to confirm the presence of causative variants identified in patients. At the same time, the initial Sanger sequencing (as the gold standard for identifying mutations) of all *SLC26A4* coding exons and adjacent splicing sites could be a successful one-step detection of all sequence variations of this gene, including those that could be considered pathogenic after a comprehensive assessment of their impact on protein structure and function. However, a thorough analysis of all required *SLC26A4* fragments can be expensive, especially in large samples of patients with unexplained HL.

This study aims to analyze the distribution of currently known causative (implicated in HL) variations in the *SLC26A4* gene for the bioinformatic preselection of its potential regions that have the most significant diagnostic value for molecular testing in patients with HL.

## 2. Results

The *SLC26A4* is a relatively large gene covering about 57 kb of genomic DNA. The canonical transcript ENST00000644269.2 (ENSEMBL: https://www.ensembl.org, accessed on 6 June 2022), which is 4737 bp long, includes 21 exons within 2343-bp cDNA, one of which (exon 1) is non-coding. Due to the large size of *SLC26A4*, there are certain difficulties in its complete mutational analysis, and it is labor-intensive, time-consuming and expensive, especially in large patient cohorts.

To select the most diagnostically significant regions of *SLC26A4*, we performed the bioinformatic analysis of the distribution of currently known causative variations in the *SLC26A4* sequence based on the data from the Deafness Variation Database (DVD: https://deafnessvariationdatabase.org/, accessed on 6 June 2022) [32]. The DVD is a comprehensive, open-access resource that integrates all available genetic, genomic, and clinical data together with expert curation to generate a single classification for each variant in the genes implicated in syndromic and non-syndromic deafness [32]. For the categorization of variants, DVD uses available information from ClinVar and/or the published literature on PubMed; an assessment of functional significance and conservation of missense variants by six computational methods (PhyloP, SIFT, LRT, MutationTaster, PolyPhen HDIV and GERP); and the data on MAF (minor allele frequency). As a result, each variant could be classified as benign (B), likely benign (LB), likely pathogenic (LP), pathogenic (P) and a variant of unknown significance (VUS). The DVD also characterizes the molecular profile of variants within different classification categories, focusing on variants in coding and splice regions, and groups them by type (nonsense, splice-site, frameshift indels, start / stop loss, in-frame indels, missense, UTRs, intronic and synonymous) across all variant classifications (P, LP, VUS, LB and B).

**Figure 1:**
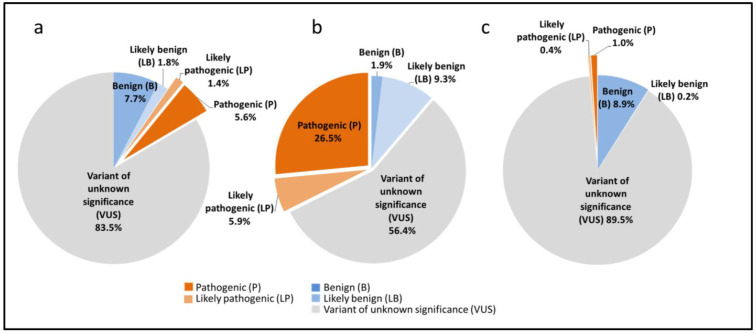
The proportions of different *SLC26A4* variants (P, LP, VUS, LB and B). (**a**) Different types of variants in the entire *SLC26A4* gene sequence. (**b**) Different types of variants in the *SLC26A4* coding region. (**c**) Different types of variants in the *SLC26A4* intronic regions.

To date, 8647 *SLC26A4* different variants (P, LP, VUS, LB and B) have been reported on the DVD (Figure 1). Among them, there are a total of 605 P and LP variants (PLP variants) and their proportions in the entire *SLC26A4* sequence and in coding and intronic regions are 7.0%, 32.4% and 1.4%, respectively (Figure 1). Only two P variants were detected in the upstream/5′-UTR regions (rs60284988/NM_000441.1:c.-103T>C and rs545973091/NM_000441.1:c.-60A>G) [33,34], while no P or LP variants were found in the 3′-UTR region.

### 2.1. PLP Variants in the SLC26A4 Coding Region

**Figure 2:**
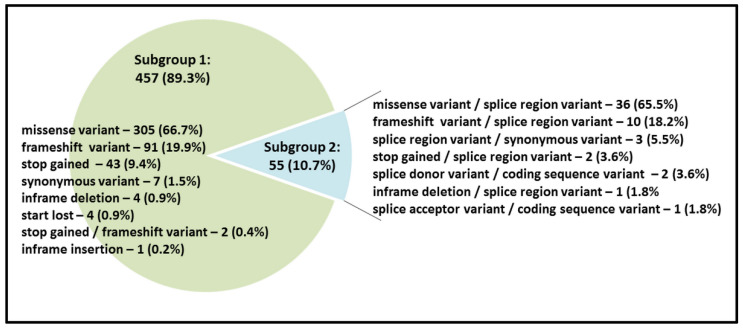
The distribution of PLP variants located in the *SLC26A4* coding region according to their molecular consequences.

The PLP variants (512 in total) make up an essential part (32.4%) of all variations in the *SLC26A4* coding region encompassing 20 exons (Figure 1b). According to their molecular consequences, the PLP variants located in the *SLC26A4* coding region can be divided into two main subgroups: (1) the variants leading to any structural changes (missense variant, frameshift variant, stop gained, inframe deletion/insertion, start lost and stop gained/frameshift variant) within the physical boundaries of the exons (457 variants, 89.3%) and (2) the variants leading to any splicing failures (55 variants, 10.7%) (Figure 2). In addition, three LP variants characterized by the DVD as the “splice acceptor variant/coding sequence variant” (c.765_765+3del and c.1539_1544+6del) or “splice donor variant/coding sequence variant” (c.919-19_932del) affect both coding and intronic *SLC26A4* regions.

Among all PLP variants in the *SLC26A4* coding region, the common molecular alterations are the missense variants, followed by the frameshift variants (Figure 2). It is worth noting that in the *SLC26A4* coding region, ten PLP variants located in different exons are synonymous (Figure 2, Table S1). These variants have only been found in individual patients with HL [23,35,36,37,38,39,40,41,42]. Currently, there is convincing evidence of the involvement of synonymous variants (“silent” genetic variations) in the development of at least 50 different human diseases; however, their pathogenic role seems to be underestimated. There is growing evidence that synonymous variants can play multiple regulatory roles in both transcription and translation (the disruption of the spliceosome, altering mRNA degradation, disruption of protein folding and the influence on the level of protein synthesis) [43,44,45]. Among ten synonymous PLP variants in the *SLC26A4* coding region, three variants, characterized by the DVD as “splice region variant/synonymous variant”, appear to result in exon skipping (Table S1). For example, according to *in silico* analysis, variant c.1803G>A (p.Lys601=), which occurs at the terminal nucleotide of exon 16, affects canonical splice donor nucleotide positions and causes the complete skipping of this exon, which was experimentally confirmed [39,42]. For other synonymous *SLC26A4* variants, their putative pathogenicity has not yet been confirmed by experimental studies.

**Figure 3:**
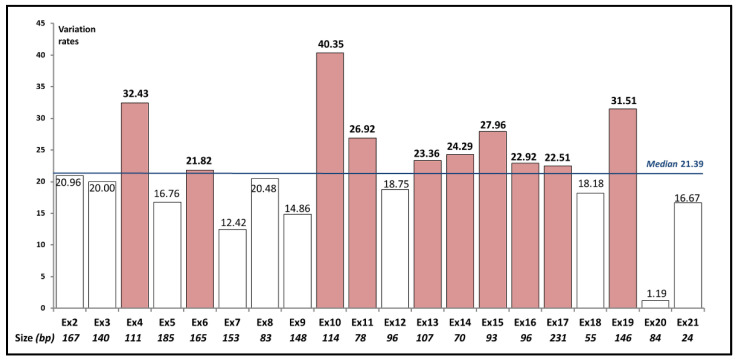
The PLP variation rates in the *SLC26A4* coding exons. The exons with the variation rates above the threshold (median = 21.39) are colored.

The physical size of *SLC26A4* coding exons varies from 55 bp (exon 18) to 231 bp (exon 17) (Ensembl: transcript: ENST00000644269.2) (Figure 3). The exception is exon 21, which comprises 2387 bp, although only initial 24 nucleotides refer to the translated sequence [46,47]. The number of known PLP variants across the *SLC26A4* coding exons also varies substantially: from 1 in exon 20 to 52 in exon 17. In order to select *SLC26A4* exons with the highest load of the PLP variants, the number of these variants was normalized by exon size (Figure 3). Ten exons (4, 6, 10, 11, 13–17 and 19) had variation rates higher than a mean (median value = 21.39) of a number of PLP variants per an exon size (Figure 3). These exons included 63.9% of all known PLP variants in the *SLC26A4* coding region.

### 2.2. PLP Variants in the SLC26A4 Intronic Regions

Knowledge on the pathogenic role of genetic variants localized in the intronic regions of genes increased significantly in recent years. Along with variants, which directly affect the splicing machinery, many studies shed light on the involvement of other intronic variants in the occurrence of many human diseases [48,49,50,51,52,53]. The “classical” splice acceptor and splice donor variants disrupting canonical splice sites at positions -1, -2 (3’ acceptor splice site) or +1, +2 (5’ donor splice site) are the most important for proper splicing and can lead to exon skipping or the emergence of an alternative splicing motif. The variants located in intronic regions near splice junctions (outside the canonical splice sites) appear to be also important for splicing, but studies that evaluated their pathogenic effects in detail on splicing are still limited [49,52,53,54]. In addition, mutations in deep intronic regions have been documented in multiple diseases. Vaz-Drago et al. [50] analyzed 185 reported deep intronic mutations across over 75 disease-associated genes. They concluded that deleterious variants located at least 100 bp from the nearest canonical splice site most commonly lead to pseudo-exon inclusion due to the activation of non-canonical splice sites or changes in splicing regulatory elements and also can disrupt transcription regulatory motifs and non-coding RNA genes [50].

**Figure 4:**
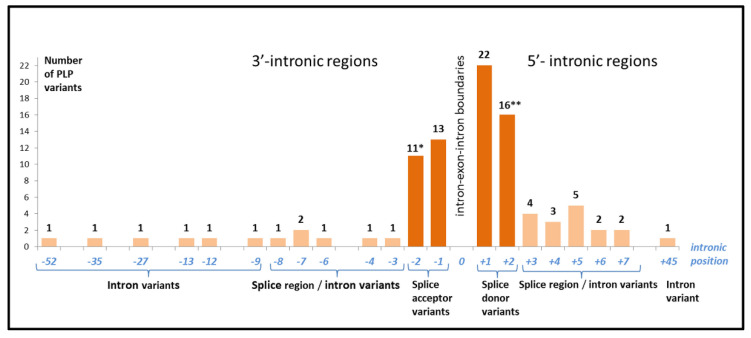
Distribution of PLP variants located in the *SLC26A4* intronic regions. *—variant c.1342-2_1343dup (splice region variant /intron variant) was included; **—variant c.164+2_164+5delinsGAGG (“splice donor variant/intron variant”) was included.

The length of the *SLC26A4* introns varies from 140 nucleotides (intron 11) to 8702 (intron 3). The PLP variants located in the *SLC26A4* intronic regions make up 1.4% of all variations in the *SLC26A4* intronic regions (Figure 1c). According to the DVD, the intronic PLP variants (91 in total) were classified as “splice donor variant” (*n* = 37), “splice acceptor variant” (*n* = 23), “splice region variant/intron variant” (*n* = 22), “intron variant” (*n* = 8), and “splice donor variant/intron variant” (*n* = 1). Among them, the “classical” splice acceptor and splice donor variants disrupting canonical splice sites at positions −1, −2 or +1, +2, are predominant (65.9%) (Figure 4). For example, a well-known pathogenic variant c.919-2A>G, which is common in Asian populations [5,7,10,19,23], is located in intron 7 and leads to skipping exon 8, with the formation of a stop codon at amino acid position 311 and, finally, a truncated form of a pendrin molecule.

Another relatively large group of intronic PLP variants (24.2% out of all PLP variants found in *SLC26A4* introns) is the “splice region variants/intron variants” group, which includes PLP variants located at −3–−8 or at +3–+7 nucleotide positions (Figure 4). These rare variants have been found in a few patients from different regions of the world. The potential damaging effects of these variants on splicing have been evaluated using different splice prediction computational tools. The experimental studies to confirm these predictions were carried out only for nine variants (c.1002-4C>G, c.1149+3A>G, c.765+3A>T, c.1001+4A>G, c.1001+5G>C, c.1001+5G>T, c.1149+3A>G, c.1341+3A>C, c.1544+5G>A and c.1707+5G>A) [42,55,56,57], although not all experimental data were consistent. For example, in the study by Park et al. [55], functional consequences of novel splice site variants c.415+4A>G and c.1707+5G>A were predicted by the BDGP Splice Site Prediction algorithm, which concluded that these variants significantly affect splicing [55]. However, later, in the study by Lee et al. [42], the assessment of these variants by minigene splicing assay revealed that variant c.1707+5G>A leads to aberrant splicing, while variant c.415+4A>G does not interrupt normal splicing [42]. Several rare PLP variants described as “intron variants” are located deeper in introns of *SLC26A4* (Figure 4). They were found in a compound heterozygosity state only in individual patients from China and Japan [8,29,35,40,58,59]. Some of them were predicted to lead to aberrant splicing [29,58,59]. A rare “splice donor variant/intron variant” c.164+2_164+5delinsGAGG in intron 2 found in only one family from Pakistan is predicted to result in skipping of exon 2 and loss of *SLC26A4* expression [60]. The “splice region variant/intron variant” 1342-2_1343dupAGTC has been reported as a compound heterozygous variant in several individuals with EVA [34]. This variant (ClinVar ID: 43506) appears to result in the duplication of a conserved splice site at the beginning of exon 12, which could either deleteriously affect the splicing of the transcript or cause a frameshift if four additional bases are included in the exon.

Thus, it can be concluded that all currently known intronic PLP variants in the *SLC26A4* gene sequence are located in close proximity (about 50 nucleotides) to exon–intron boundaries, thereby determining the importance of these *SLC26A4* regions for diagnostic purposes.

### 2.3. Prevalence of the SLC26A4 PLP Variants in Different Populations

One of the important criteria for the variant interpretation used by the DVD is the information about their MAFs (minor allele frequencies) obtained from the Genome Aggregation Database (gnomAD, https://gnomad.broadinstitute.org/, accessed on 6 June 2022). In most genes associated with recessive HL, variants with a MAF of ≥0.005 (0.5%) are likely to be benign. However, there are certain exceptions and limitations because several genes, including *SLC26A4,* do not adhere to this cutoff; in small populations, certain variants may be observed with higher MAFs; in addition, MAFs are not available for all variants [32,61].

The data on MAFs for PLP variants of the *SLC26A4* gene presented on the DVD, which were obtained from gnomAD (version 2.1.1), are currently available for only 33.9% (205/605) of them. The MAFs of the *SLC26A4* PLP variants vary from 0% (three variants p.Ser93ArgfsTer4, p.Pro123Ser and p.Gln736Ter) to 0.506367% (variant c.919-2A>G, common in East Asian populations). Ultra-rare (0% < MAF% < 0.05%) variants prevail and represent 85.4% (*n* = 175/205) of all PLP variants with available MAFs while the MAFs for the remaining 27 PLP variants exceed 0.05%.

Another set of data on the prevalence of pathogenic *SLC26A4* variants can be obtained from numerous studies aimed at assessing the contribution of *SLC26A4* to the HL etiology and studying the genotype–phenotype correlations mediated by the presence of different *SLC26A4* pathogenic variants in patients. Lu et al. [19] performed a comprehensive meta-analysis to evaluate the diagnostic value of *SLC26A4* mutant alleles and their correlations with multiethnic hearing phenotypes in EVA patients [19]. They summarized data from 24 articles from Asia, Europe and North America (2294 cases and 3193 controls in multiethnic cohorts) and revealed a set of *SLC26A4* mutations (*n* = 22) that were at the top of 10% mutation rate in patients with HL in all ethnicities [19].

To select the most frequent PLP variants in the *SCL26A4* gene, we integrated the data on the allelic frequencies of common *SLC26A4* mutations from the study by Lu et al. [19] with the PLP variants having the highest MAFs (not less than 0.05%) from the data on the gnomAD populations (presented on the DVD). Summarized data including 42 PLP variants are presented in Table S2. Twenty PLP variants with MAF > 0.05% were not mentioned in the list of common *SLC26A4* mutations from the study by Lu et al. [19]. High *SLC26A4* mutation rates in patients and relatively high MAFs (>0.05%) coincided for only seven common *SLC26A4* mutations: c.919-2A>G, p.His723Arg and p.Ala360Val in East Asian populations (gnomad_AF_eas); p.Phe335Leu and p.Thr410Met in South Asian populations (gnomad_AF_sas); p.Gly209Val and p.Leu236Pro in European (non-Finnish) populations (gnomad_AF_nfe). Among other 13 common *SLC26A4* mutations from the study by Lu et al. [19], the MAF varies from 0 (variants p.Ala372Val and p.Leu676Gln) to 0.0397655% (variant c.1001+1G>A) (Table S2).

## 3. Discussion

Screening for *SLC26A4* pathogenic variants in patients with HL is an important part of molecular genetic testing for HL. Understanding the rates of *SLC26A4*-related HL in a particular population is very important to develop an optimal algorithm for molecular testing. However, despite the fact that such data are already known from numerous studies [5,6,7,9,17,18,19,20], there is no accurate evaluation of the pathogenic contribution of *SLC26A4* to HL in different regions of the world.

The phenotypes of *SLC26A4*-related HL range from nonsyndromic recessive sensorineural HL (DFNB4) to Pendred syndrome with varying onsets with respect to its full clinical manifestation. In addition to this, the enlargement of the vestibular aqueduct (EVA) is a penetrant feature of *SLC26A4*-related HL. All of this predetermines the heterogeneity of the examined cohorts of patients (patients with nonsyndromic HL without the preselection of EVA or Pendred syndrome, patients with diagnosed EVA or Pendred syndrome, pediatric or adult patients, etc.) in different studies. In addition, a search for casual variants in *SLC26A4* during routine molecular diagnostics is complicated by the large size of this gene, including 20 coding exons and diagnostically important flanking intronic regions.

Various strategies, including a targeted search of particular pathogenic *SLC26A4* variants, different multi-step hierarchical screenings and/or the NGS techniques, are currently used for the molecular diagnostics of the *SLC26A4*-related HL; however, their diagnostic rates can vary depending on the applied methods [6,21,22,23,24,25,26,27,28,29,30,31]. All of these diagnostic approaches have certain limitations, such as, for example, the possible omission of other pathogenic *SLC26A4* variants in targeted screenings and the still limited use of NGS analysis for routine diagnostics. In this regard, it seems important to optimize the testing of the *SLC26A4* gene by the preliminary selection of the most diagnostically important regions of this gene for their priority analysis. A thorough analysis of the distribution of known causal variations along the *SLC26A4* gene sequence followed by a selection of its regions with the highest load of such variants seems reasonable to solve this problem.

We selected all known PLP variants in the *SLC26A4* gene currently reported in the DVD (605 in total) and analyzed their distributions along the sequence of this gene. The PLP variants located within the physical boundaries of *SLC26A4* coding exons account for about 85% (512 in total) of all PLP variants in this gene. Most of them directly lead to any changes in the structure and functions of the protein (missense, frameshift, stop gained and start lost variants); however, some of such variants (*n* = 55) are known to affect the proper splicing process. All currently known intronic PLP variants (91 in total) in the *SLC26A4* gene sequence are located in close proximity (about 50 nucleotides) to the exon-intron boundaries and disrupt splicing. Most are canonical splice sites (−1, −2 or +1, +2) variants. Thus, in total, PLP variants that in any way affect splicing make up a significant part of all PLP variants in the *SLC26A4* gene (24.1%, 146/605).

**Figure 5:**
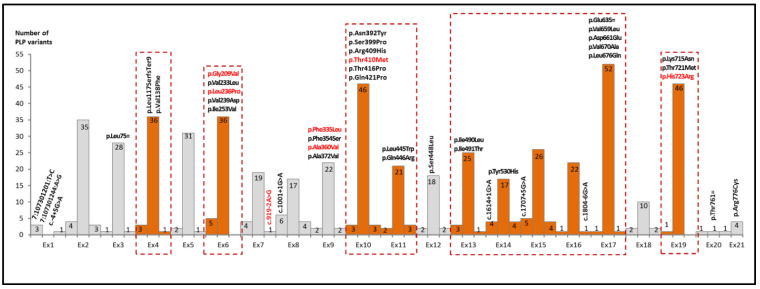
The regions of the *SLC26A4* gene sequence with the highest diagnostic value. The exons with high rates of the PLP variants with adjacent intronic regions are marked in orange color and highlighted with a dotted line. The most common PLP variants (*n* = 42, see in text and Table S2) are shown above the bars. Seven variants with both relatively high MAFs (>0.05%) and high mutation rates in patients are shown in red.

In order to identify the *SLC26A4* regions with the highest load of PLP variants, we combined the data on PLP variants located in coding exons with those in adjacent intronic regions (about 50 nucleotides). As a result, ten regions of the *SLC26A4* gene sequence (exons 4, 6, 10, 11, 13–17 and 19 and their flanking intronic regions), which appear to have the greatest potential diagnostic value, were identified (Figure 5). Mutational analysis of these parts of *SLC26A4* gene sequence can potentially provide a diagnostic rate of 61.9% of all currently known PLP variants in this gene.

The increased rates of PLP variants in several specific regions of the *SLC26A4* gene sequence may indicate that they affect the most functionally significant domains of the pendrin protein. No experimental structure of pendrin is currently available, and different topology models with varying numbers (from 11 to 15) of transmembrane segments were proposed [62,63,64]. Pendrin belongs to the SLC26 anion transporter protein family. The SLC26 proteins were originally predicted to contain ~12 transmembrane (TM) segments with the cytoplasmic N- and C-termini followed by the STAS (Sulphate Transporter and AntiSigma factor antagonist) domain at the C-terminus [64,65,66]. The presence of disease-causing mutations in the STAS domains of other SLC26 proteins attests to the structural importance of this functional region of pendrin, although the exact role of the STAS domain has not yet been elucidated [65,66]. It has been suggested that the STAS domain may play a role in nucleotide binding and/or interactions with other proteins, including other transporters, cytoskeletal scaffolds and with enzymes metabolizing transported anion substrates [62,66,67].

The STAS domain of the pendrin protein consists of 195 amino acid residues (from 535/536 to 728/729 amino acid positions) encoded by exons 14–19 (UniProt: https://www.uniprot.org/uniprot/O43511, accessed on 6 June 2022); Deafness Variation Database: https://deafnessvariationdatabase.org/gene/SLC26A4, accessed on 6 June 2022). About one-quarter of all PLP variants in the *SLC26A4* coding exons are located in this particular *SLC26A4* region, thereby emphasizing its diagnostic value.

The data on the prevalence of *SLC26A4* PLP variants in different regions of the world are very important for the development of optimal algorithms for the diagnostic testing of *SLC26A4* in a particular population. Currently, the general trends in the worldwide prevalence of different *SLC26A4* PLP variants have not yet been fully elucidated. The uneven prevalence of PLP variants in the *SLC26A4* gene in different populations or geographical regions may be influenced by the ethnicity of the studied patients, the history of a population to which they belong, and by the heterogeneity of the examined cohorts of patients or a variable sensitivity of diagnostic methods applied in a particular group of patients.

The prevalence of *SLC26A4* PLP variants in different regions of the world was presented in several reviews [6,8,9,18,19,20,40,68]. The presence of relatively few *SLC26A4* PLP variants (p.Arg409His, p.Thr410Met, p.Leu445Trp, and p.Leu597Ser) can be ubiquitously observed in all regions of the world; however, their frequencies vary greatly depending on the specific region [9,19,20,68]. The spectrum of *SLC26A4* pathogenic variants found in Asian populations appears to differ from that in populations of Caucasian origin. At the very least, the variants c.919-2A>G and p.His723Arg are the most common in East Asian populations, while they are very rare in Europe; by contrast, variants c.1001+1G>A, p.Val138Phe, p.Thr416Pro, p.Leu236Pro and p.Gly209Val are prevalent in many Caucasian populations [9,19].

The accumulation of specific PLP variants in certain populations can be a result of the founder effect, as it was evidenced for p.His723Arg in Japanese and Koreans and c.919-2A>G in Chinese [5,9,69]. The role of founder effect was also suggested in the prevalence of several other PLP variants in some local populations: p.Ser90Leu, p.Val239Asp, p.Gln446Arg in families from Pakistan [68,70], p.Val138Phe in German patients [71], c.965dup (p.Asn322LysfsTer8) in Iranian patients [72] and p.Gln514Arg in Spanish patients [36]. In addition, in our recent study [73], we revealed high frequencies of variant p.Leu676Gln, which is rare in other populations, and of a novel variant c.1545T>G (p.Phe515Leu), which both explain a significant part of the *SLC26A4*-related HL in Tuvinian patients belonging to indigenous Turkic-speaking people living in the Tyva Republic (Southern Siberia, Russia).

The integration of data on PLP variants with relatively high MAFs (>0.05%) derived from the gnomAD populations with data on allelic frequencies of the most frequent *SLC26A4* mutations in patients worldwide from a meta-analysis by Lu et al. [19] allowed us to identify 42 most common PLP variants, although high MAFs and high mutation rates in patients have coincided for only seven of them (Table S2, Figure 5).

Thus, the current data on the distribution of *SLC26A4* PLP variants in different populations do not allow making a reasonable conclusion about general patterns of their prevalence worldwide.

Mutational analysis of the *SLC26A4* gene in a particular patient, due to a recessive inheritance pattern of the *SLC26A4*-related HL, should continue until two recessive pathogenic variants of *SLC26A4* are detected and, thereby, molecular diagnosis could be made. Various strategies are used to diagnose the *SLC26A4*-associated HL. Each of the diagnostic methods has its own advantages and limitations, but in any case, direct Sanger sequencing (as the gold standard for identifying mutations) is considered necessary to confirm the presence of causative variants that are identified in patients. The priority sequencing of the most diagnostically important regions of the *SLC26A4* gene, selected in our study, could be a successful one step-detection of 61.9% of all currently known PLP variants in the *SLC26A4* gene. This approach can be effective as a relatively inexpensive initial diagnostic testing in large samples of patients of unknown ethnicity or it can be applied in any multi-step hierarchical diagnostic screenings, specifically designed for a particular population or region. For example, in the case when it is already known that some specific PLP variants are most common in a certain population (c.919-2A>G in Chinese or p.His723Arg in Japanese and Koreans), this approach can also be applied after a targeted analysis of such variants.

## 4. Materials and Methods

For the bioinformatic analysis of *SLC26A4* gene sequence variations, we used the DVD v9 version of the Deafness Variation Database (DVD), which includes all known genetic variants presented in 224 deafness-associated genes (Deafness Variation Database: https://deafnessvariationdatabase.org/gene/SLC26A4, accessed on 6 June 2022) [32].

**Web Resources:**

***SLC26A4 gene:***

The canonical transcript: ENST00000644269.2 (ENSEMBL: https://www.ensembl.org/, accessed on 6 June 2022).

NCBI Reference Sequence:

NM_000441.2 (https://www.ncbi.nlm.nih.gov/nuccore/NM_000441, accessed on 6 June 2022),

NC_000007.13 (https://www.ncbi.nlm.nih.gov/nuccore/NC_000007.13, accessed on 6 June 2022).

The consensus coding sequence (CCDS): CCDS 5746.1. (https://www.ncbi.nlm.nih.gov/projects/CCDS/CcdsBrowse.cgi?REQUEST=ALLFIELDS&DATA=CCDS5746, accessed on 6 June 2022).

***Protein pendrin:***

NCBI Reference Sequence: NP_000432.1 (https://www.ncbi.nlm.nih.gov/protein/NP_000432.1, accessed on 6 June 2022).

UniProt: O43511 (UniProt: https://www.uniprot.org/uniprot/O43511, accessed on 6 June 2022).

## 5. Conclusions

The bioinformatic analysis of the distribution of PLP variants across the *SLC26A4* gene sequence revealed the most diagnostically important regions of this gene (exons 4, 6, 10, 11, 13–17 and 19 with flanking intronic regions), the sequencing of which can provide a diagnostic rate of 61.9% for all currently known PLP variants in the *SLC26A4* gene. The primary analysis of these *SLC26A4* regions may be applied as an initial effective diagnostic test in samples of patients of unknown ethnicity or as a subsequent step after the targeted testing of already-known ethno- or region-specific pathogenic *SLC26A4* variants.

## Data Availability

The data presented in this study are available in this article and in Supplementary Materials.

## Supplementary Materials

The following supporting information can be downloaded at: https://www.mdpi.com/article/10.3390/ijms232113453/s1.
