# Supplementary material for: Selection of Diagnostically Significant Regions of the SLC26A4 Gene Involved in Hearing Loss
Source: Int J Mol Sci. 2022 Nov 3;23(21):13453. doi: 10.3390/ijms232113453 (PMC9655724; doi:10.3390/ijms232113453)
Supplement: Supplementary file 1 [file ijms-23-13453-s001.zip › Table S1.pdf]

Table S1. The synonymous variants in the *SLC26A4* coding region.

Genomic Description (GRCh37)	HGVS Nucleotide Change (NM_000441.2)	HGVS Protein Change (NP_000432.1)	Variant Classification	Consequence	Exon	References
7:107303801:C>G	c.225C>G	p.Leu75=	P	Synonymous variant	3/21	[23,35–37,40]
7:107315467:T>C	c.678T>C	p.Ala226=	P	Synonymous variant	6/21	[23,35,37]
7:107323799:G>A	c.918G>A	p.Val306=	LP	Splice region variant & synonymous variant	7/21	-
7:107330643:C>T	c.1224C>T	p.Ser408=	P	Synonymous variant	10/21	-
7:107334901:G>C	c.1317G>C	p.Gly439=	LP	Synonymous variant	11/21	[38]
7:107341641:G>A	c.1803G>A	p.Lys601=	P	Splice region variant & synonymous variant	16/21	[39,42]
7:107342373:G>A	c.1905G>A	p.Glu635=	P	Synonymous variant	17/21	[23,35,37]
7:107342502:G>A	c.2034G>A	p.Val678=	LP	Splice region variant & synonymous variant	17/21	[41]
7:107350614:T>G	c.2205T>G	p.Ser735=	P	Synonymous variant	19/21	[35]
7:107353031:A>G	c.2283A>G	p.Thr761=	P	Synonymous variant	20/21	[35,40]
