# Supplementary material for: Selection of Diagnostically Significant Regions of the SLC26A4 Gene Involved in Hearing Loss
Source: Int J Mol Sci. 2022 Nov 3;23(21):13453. doi: 10.3390/ijms232113453 (PMC9655724; doi:10.3390/ijms232113453)
Supplement: Supplementary file 1 [file ijms-23-13453-s001.zip › Table S2.pdf]

Table S2. Combined data on the prevalence of most frequent PLP variants in the *SLC26A4* gene.

	Variant	Data from the DVD v9 version *					Data from [19]
		Exon	Intron	Variant Classification	Max MAF (%)	Max MAF Source	Allele frequency in cohorts of patients
1	7:107301201:T>C	null	null	P	0.337137	gnomad_AF_nfe_pass	-
2	7:107301244:A>G	null	null	P	0.149254	gnomad_AF_afr_pass	-
3	c.-4+5G>A	null	1/20	LP	0.235849	gnomad_AF_amr_pass	-
4	p.Leu75= (c.225C>G)	3/21	null	P	0.0952954	gnomad_AF_eas_pass	-
5	p.Leu117SerfsTer9 (c.349del)	4/21	null	P	0.0641026	gnomad_AF_eas_pass	-
6	p.Val138Phe (c.412G>T)	4/21	null	P	0.0294542	gnomad_AF_nfe_pass	0.098
7	p.Gly209Val (c.626G>T)	6/21	null	P	0.0580729	gnomad_AF_nfe_pass	0.070
8	p.Val233Leu (c.697G>C)	6/21	null	P	0.14135	gnomad_AF_eas_pass	-
9	p.Leu236Pro (c.707T>C)	6/21	null	P	0.0596418	gnomad_AF_nfe_pass	0.039
10	p.Val239Asp (c.716T>A)	6/21	null	P	0.16658	gnomad_AF_sas_pass	-
11	p.Ile253Val (c.757A>G)	6/21	null	LP	0.0598021	gnomad_AF_eas_pass	-
12	c.919-2A>G	null	7/20	P	0.506367	gnomad_AF_eas_pass	0.624
13	c.1001+1G>A	null	8/20	P	0.0397655	gnomad_AF_nfe_pass	0.045
14	p.Phe335Leu (c.1003T>C)	9/21	null	P	0.248269	gnomad_AF_sas_pass	0.037
15	p.Phe354Ser (c.1061T>C)	9/21	null	P	0.149658	gnomad_AF_amr_pass	-
16	p.Ala360Val (c.1115C>T)	9/21	null	P	0.0641026	gnomad_AF_eas_pass	0.058
17	p.Ala372Val (c.1115C>T)	9/21	null	P	0	null	0.064
18	p.Asn392Tyr (c.1174A>T)	10/21	null	P	0.00543833	gnomad_AF_eas_pass	0.039
19	p.Ser399Pro (c.1195T>C)	10/21	null	P	0.0881892	gnomad_AF_sas_pass	-
20	p.Arg409His (c.1226G>A)	10/21	null	P	0.0197684	gnomad_AF_amr_pass	0.072
21	p.Thr410Met (c.1229C>T)	10/21	null	P	0.0587928	gnomad_AF_sas_pass	0.066
22	p.Thr416Pro (c.1246A>C)	10/21	null	P	0.0365452	gnomad_AF_nfe_pass	0.062
23	p.Gln421Pro (c.1262A>C)	10/21	null	LP	0.00501505	gnomad_AF_eas_pass	0.047
24	p.Leu445Trp (c.1334T>G)	11/21	null	P	0.0186616	gnomad_AF_nfe_pass	0.089
25	p.Gln446Arg (c.1337A>G)	11/21	null	LP	0.0588005	gnomad_AF_sas_pass	-
26	p.Ser448Leu (c.1343C>T)	12/21	null	LP	0.0108814	gnomad_AF_eas_pass	0.041
27	p.Ile490Leu (c.1468A>C)	13/21	null	P	0.24497	gnomad_AF_sas_pass	-
28	p.Ile491Thr (c.1472T>C)	13/21	null	P	0.0601443	gnomad_AF_eas_pass	-
29	p.Tyr530His (c.1588T>C)	14/21	null	P	0.00440319	gnomad_AF_nfe_pass	0.091
30	c.1614+1G>A	null	14/20	P	0.00352541	gnomad_AF_nfe_pass	0.056
31	c.1707+5G>A	null	15/20	P	0.0109206	gnomad_AF_eas_pass	0.042
32	c.1804-6G>A	null	16/20	P	0.29096	gnomad_AF_eas_pass	-
33	p.Glu635= (c.1905G>A)	17/21	null	P	0.160417	gnomad_AF_eas_pass	-
34	p.Val659Leu (c.1975G>C)	17/21	null	P	0.0200562	gnomad_AF_eas_pass	0.036
35	p.Asp661Glu (c.1983C>A)	17/21	null	P	0.165463	gnomad_AF_eas_pass	-
36	p.Val670Ala (c.2009T>C)	17/21	null	P	0.0902437	gnomad_AF_eas_pass	-
37	p.Leu676Gln (c.2027T>A)	17/21	null	P	0	null	0.049
38	p.Lys715Asn (c.2145G>T)	19/21	null	P	0.0947403	gnomad_AF_sas_pass	-
39	p.Thr721Met (c.2162C>T)	19/21	null	P	0.0289503	gnomad_AF_amr_pass	0.041
40	p.His723Arg (c.2168A>G)	19/21	null	P	0.160385	gnomad_AF_eas_pass	0.261
41	p.Thr761= (c.2283A>G)	20/21	null	P	0.145334	gnomad_AF_eas_pass	-
42	p.Arg776Cys (c.2326C>T)	21/21	null	LP	0.289631	gnomad_AF_nfe_pass	-

* – Deafness Variation Database: https://deafnessvariationdatabase.org/gene/*SLC26A4*, accessed on 6 June 2022), NC_000007.13 (https://www.ncbi.nlm.nih.gov/nuccore/NC_000007.13, accessed on 6 June 2022), NM_000441.2 (https://www.ncbi.nlm.nih.gov/nuccore/NM_000441.2, accessed on 6 June 2022), NP_000432.1 (https://www.ncbi.nlm.nih.gov/protein/NP_000432.1, accessed on 6 June 2022).

Seven *SLC26A4* PLP variants with coinciding high MAFs and high mutation rates in patients are highlighted in grey.
